# Aesthetic result after breast-conserving therapy is associated with quality of life several years after treatment. Swedish women evaluated with BCCT.core and BREAST-Q™

**DOI:** 10.1007/s10549-017-4306-5

**Published:** 2017-05-23

**Authors:** Cecilia Dahlbäck, Jenny Heiman Ullmark, Martin Rehn, Anita Ringberg, Jonas Manjer

**Affiliations:** 1 Department of Surgery, Skåne University Hospital, Malmö, Sweden; 2 Department of Clinical Sciences, Malmö, Lund University, Lund, Sweden; 3 Department of Surgery, Sahlgrenska University Hospital, Gothenburg, Sweden; 4 Institute of Clinical Sciences, Sahlgrenska Academy at the University of Gothenburg, Gothenburg, Sweden; 5 Department of Plastic and Reconstructive Surgery, Skåne University Hospital, Malmö, Sweden

**Keywords:** Breast-conserving therapy, BCCT.core, BREAST-Q, Aesthetic result, Health-related quality of life

## Abstract

**Purpose:**

A gold standard for evaluation of aesthetic outcome after breast-conserving therapy (BCT) is still lacking. The BCCT.core software has been developed to assess aesthetic result in a standardised way. We aimed to study how the result of BCCT.core after BCT is associated with quality of life, measured with the BREAST-Q™, a validated questionnaire.

**Methods:**

Women eligible for BCT were consecutively recruited between February 1st 2008 and January 31st 2012 (*n* = 653). Photographs of 310 women, taken one year after BCT, were evaluated using the BCCT.core software. The postoperative BCT module of the BREAST-Q™ questionnaire was administered by mail and 348 questionnaires were returned (median 5.5 years after BCT). In all, 216 women had both BCCT.core results and completed BREAST-Q™ questionnaires available.

**Results:**

The results from the BCCT.core evaluation were: excellent *n* = 49 (15.8%); good *n* = 178 (57.4%); fair *n* = 73 (23.5%); poor *n* = 10 (3.2%). The median BREAST-Q™ score for satisfaction with breasts was 66 [interquartile range (IQR) 57–80] and for psychosocial well-being 82 (IQR 61–100). Poor/fair results on BCCT.core were associated with *Q*-scores below median for both satisfaction with breasts [odds ratio (OR) 3.4 (confidence interval (CI) 1.7–6.8)] as well as for psychosocial well-being [OR 2.2 (CI 1.1–4.2)].

**Conclusions:**

A statistically significant association between BCCT.core results one year after BCT and quality of life ratings using BREAST-Q™ several years later is shown in this study. This implies that the BCCT.core may be valuable in BCT follow-up and used as a standardised instrument in the evaluation of aesthetic results.

## Introduction

Breast cancer is the most common cancer in women in Sweden, with an annual incidence of approximately 160 per 100,000 individuals [1]. About 75% [2] of women with an unifocal breast cancer up to three centimetres in diameter are treated with breast-conserving therapy (BCT), i.e., a partial mastectomy followed by adjuvant radiotherapy, which has been shown to have equal mortality rates compared to mastectomy when treating small breast tumours [3]. The great majority of patients survive treatment long term [2], making postoperative aesthetic outcome and health-related quality of life (HRQoL) important outcome measures. HRQoL can be assessed by patient-reported outcome measures (PROMs). For evaluation of PROM in women undergoing breast surgery, a validated questionnaire, i.e., the BREAST-Q™ has been developed [4–10]. The BREAST-Q™ BCT module has recently been translated into Swedish.

Much research has been done to assess the aesthetic outcome after breast cancer surgery [11–14]. However, comparisons between studies are difficult since standardised instruments are lacking. A research group in Portugal (INESC porto research group), aiming to create an objective tool to evaluate breast aesthetics, has developed a software, i.e., the Breast Cancer Conservative Treatment. cosmetic results (BCCT.core). Assessing two-dimensional photographs, BCCT.core produces a rated result: excellent, good, fair, or poor aesthetic outcome, based on symmetry, skin colour, and scar appearance [15, 16].

The aim of this study was to investigate how the postoperative aesthetic result, evaluated with the BCCT.core approximately a year after BCT, correlated with the patients’ HRQoL after additional follow-up time had elapsed, using the BREAST-Q™ questionnaire.

## Methods

### Patients

**Fig. 1:**
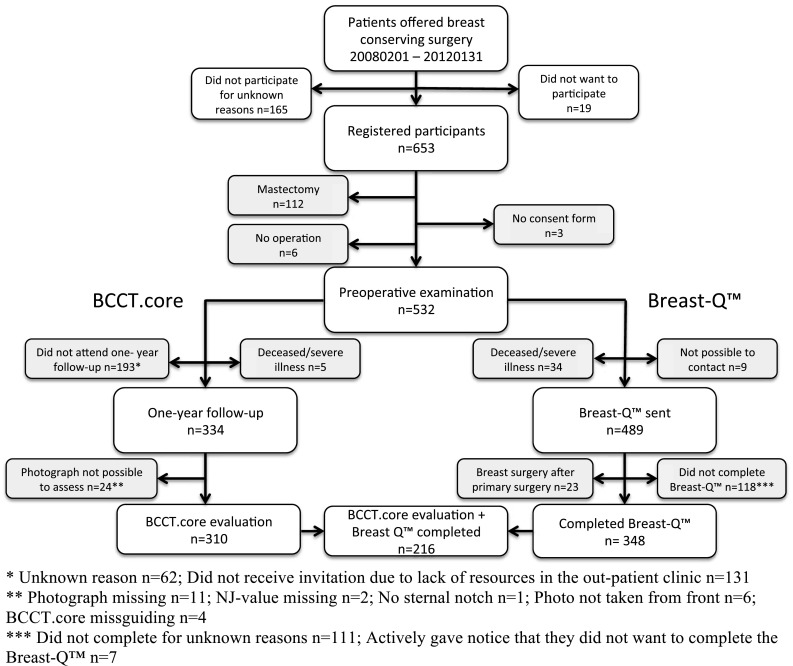
Study population

Patients who were offered BCT due to a suspected breast cancer, at Skåne University Hospital Malmö, between February 1st 2008 and January 31st 2012, were eligible for inclusion in the study. Comprehension of given information in spoken and written Swedish was warranted. The patients were usually offered BCT if the tumour was unifocal, less than four centimetres in diameter, and if the surgeon considered that the postoperative aesthetic result would be acceptable. A total of 653 patients were identified as eligible participants and were registered in the study database (Fig. 1). The material was retrospectively compared to the National Swedish Breast Cancer Registry. It was established that 78% of potential participants had been registered [17].

### Baseline examination

The attending surgeon did the preoperative examination. Age at the time of surgery was registered. Weight was measured in kilograms (one decimal) and height to the closest half centimetre. Breast volume in millilitres was measured bilaterally by use of plastic cups, specially designed and previously validated [18, 19]. Nipple to jugular notch (NJ) distance was measured bilaterally to the closest half centimetre.

### Surgery and adjuvant treatment

The surgical technique used was registered by the surgeon. Mobilisation of the breast tissue from the overlying skin and pectoral fascia was routine in a “conventional” partial mastectomy, which was performed in 503 cases. Oncoplastic breast surgery techniques were registered in 29 cases. Reduction mammaplasty of the opposite breast to achieve symmetry was performed in seven cases. Five patients had bilateral tumours. Six breast surgeons performed 99% of the operations.

The sentinel node technique was routinely used for examination of nodal involvement of the axilla. If metastases were found, a completing axillary lymph node dissection was performed. Specimen weight in grams was compared to the preoperative breast volume to assess the estimated percentage of breast volume excised (EPBVE). Chemotherapy, radiotherapy, and hormonal treatment were given according to national guidelines [20]. Depending on patient age and tumour characteristics adjuvant radiotherapy was administered to the remaining breast parenchyma. Twenty-five women were enrolled in a parallel ongoing study, which evaluated oncological outcome after breast-conserving surgery (BCS) without radiotherapy in women with age >65 treated with hormonal therapy.

### Follow-up

Approximately one year after completed radiotherapy, patients were invited to follow-up. A trained nurse photographed the patients from the front with arms down (frontal projection) using a using a Nikon Coolpix S200 camera. Body weight, breast volumes, and NJ-distances were measured. During certain time periods, invitations to follow-up were not sent out as planned, due to limited resources in the out-patient clinic. This accounted for 131 of the 193 non-attending patients. The follow-up visit took place after a median of 16 months [interquartile range (IQR) 15–18; range 11–23] after the operation.

### BCCT.core

The postoperative photo (frontal projection) was evaluated using the BCCT.core software. According to the BCCT.core manual, a marker is to be placed 25 cm down from the jugular notch to calibrate distance. Since the NJ-distance had been recorded at the same time that the patient was photographed, this calibration could be performed in retrospect. Using Gimp^©^ software (GNU image manipulation program), a free and open source image editor on the Internet, the NJ-distance was measured in the photo, divided by the actual NJ-distance recorded at the visit, and the result was subsequently multiplied by 25, resulting in a calibrated distance to be marked out. The photo was evaluated in the BCCT.core software, obtaining a result: excellent, good, fair, or poor. The software and the algorithms for evaluation are available in an article by Cardoso et al. [15].

### BREAST-Q™

The BREAST-Q™ is a disease-specific validated questionnaire for evaluating PROM. It was developed by the Memorial Sloan Kettering Cancer Institute and the University of British Columbia.^1^ The BCT module was developed for BCS with adjuvant radiotherapy, i.e., BCT for breast cancer.

In 2015, the translation of the BCT module to Swedish was completed. The translation process followed the “linguistic validation of a patient-reported outcomes measure” guidelines provided by the MAPI Research Trust. The original questionnaire (source language) was subjected to a “forward translation” by two professional translators, creating their own independent Swedish translation. These two translations were compared and combined into an accepted translation (version 1). A professional translator then subjected this version to a “backward translation” into the source language (version 2) without access to the source document. Version 2 was compared to the source document and the Swedish version (version 1) was adjusted into a final translated version (version 3). Version 3 of the pre- and postoperative questionnaire was subjected to face-validation on the appropriate patient group. The preoperative group consisted of five women scheduled for breast cancer surgery the next day. The age span of these women was 36–64 years. The postoperative group consisted of five women, age 52–68 years, previously subjected to BCT. An experienced research nurse conducted the face validation. Following some minor adjustments to version 3, the final Swedish translation was approved.

The postoperative BREAST-Q™ questionnaire was administered to 489 women by mail together with an explanatory letter and a study-specific questionnaire. Two reminders were sent by mail to those who had not returned the questionnaire. Following this, the response rate was 76%. The women were asked to report when they last had surgery to either breast, and 23 women who had undergone additional breast surgery after the one-year follow-up were excluded. In all, 348 BREAST-Q™ questionnaires were available for evaluation. The BREAST-Q™ was completed with a median of 5.5 years postoperatively (range 3.7–7.9 years).

### Statistical methods

The association between results of the BCCT.core and different potential determinants was analysed with cross tables and statistical significance tested using a χ^2^ test. *p* values below 0.05 were considered statistically significant. Continuous variables among the potential determinants were categorised into subgroups.

The result from the BREAST-Q™ postoperative questionnaire was analysed according to the user instructions provided by the MAPI Research Trust. Patients who had answered less than half of the questions in a domain were excluded from analysis of that specific domain. The resulting score for each domain was converted to a *Q*-score (range 0–100) by the use of a manual scoring table, as recommended by the MAPI Research Trust.

When comparing *Q*-scores to the results of the BCCT.core, *Q*-scores were divided into four groups based on quartiles. In an additional analysis, the effect of the BCCT.core result on the result of BREAST-Q™ was investigated. To enable the use of logistic regression analysis, *Q*-scores were dichotomised with a cut-off based on the median value. Using logistic regression, odds ratios (OR) were obtained, with 95% confidence intervals (CI). The BCCT.core result was here presented both in the original four groups but also dichotomised into “excellent/good” and “fair/poor” to simplify the interpretation. The results were adjusted for age and in an additional model, for factors that in the univariate analysis showed a statistically significant association with the BCCT.core result.

Statistical analysis was performed using IBM SPSS^®^ Statistics for Macintosh, Version 22.0. Armonk, NY: IBM Corp.

## Results

Preoperative characteristics of all included patients (*n* = 532), those with BCCT.core evaluation available (*n* = 310), those with BREAST-Q™ questionnaires available (*n* = 348) and those with both (*n* = 216) are presented in Table 1. The characteristics in the different groups are very similar.

**Table 1:** Preoperative characteristics

	Patients included (*n* = 532)	BCCT.core evaluated (*n* = 310)	Breast-Q™ completed (*n* = 348)	BCCT.core and Breast-Q™ (*n* = 216)
Age (years)	60 (51–67)^a^	62 (54–68)	60 (51–67)	61 (54–67)
BMI (kg/m^2^)	26 (23–29)	25 (23–29)	26 (23–30)	25 (23–29)
Breast size (ml)	500 (375–800)	500 (375–790)	500 (375–825)	500 (375–737.5)
Tumour size (mm)	15 (10–20)	15 (10–20)	15 (10–20)	15 (10–20)

^a^median (IQR)

Results of the BCCT.core were: excellent *n* = 49 (15.8%); good *n* = 178 (57.4%); fair *n* = 73 (23.5%); poor *n* = 10 (3.2%). Associations with potential determinants are shown in Table 2, along with *p* values. BMI, preoperative breast size and radiotherapy each had a statistically significant association with the resulting score. Women with low BMI (<25) and smaller breasts preoperatively scored better on the BCCT.core, whereas radiotherapy was associated with poorer results.

**Table 2:** BCCT.core score in relation to potential determinants

Factor	Poor	Fair	Good	Excellent	*p* value
Age at operation (years)					
<50	0 (0)	10 (13.7)	29 (16.3)	10 (20.4)	0.398
≥50–<65	4 (40.0)	27 (37.0)	82 (46.1)	19 (38.8)	
≥65	6 (60.0)	36 (49.3)	67 (37.6)	20 (40.8)	
BMI at operation (kg/m^2^)					
<25	2 (20.0)	24 (32.9)	77 (43.3)	29 (59.2)	0.002
≥25–<30	6 (60.0)	22 (30.1)	68 (38.2)	9 (18.4)	
≥30	2 (20.0)	27 (37.0)	33 (18.5)	10 (20.4)	
Missing				1 (2.0)	
Breast size preoperatively (ml)					
<450	1 (10.0)	17 (23.6)	57 (32.6)	21 (43.8)	0.019
≥450–<650	5 (50.0)	18 (25.0)	62 (35.4)	15 (31.3)	
≥650	4 (40.0)	37 (51.4)	56 (32.0)	12 (25.0)	
Estimated percentage of breast volume excised (%)					
<10	2 (20.0)	20 (27.4)	52 (29.2)	18 (36.7)	0.331
≥10–<20	4 (40.0)	36 (49.3)	92 (51.7)	22 (44.9)	
≥20	4 (40.0)	13 (17.8)	23 (12.9)	6 (12.2)	
Missing	0 (0)	4 (5.5)	11 (6.2)	3 (6.1)	
Oncoplastic breast surgery					
No	10 (100)	69 (94.5)	165 (92.7)	46 (93.9)	0.795
Yes	0 (0)	4 (5.5)	13 (7.3)	3 (6.1)	
Axillary clearance					
No	9 (90.0)	55 (75.3)	147 (83.1)	43 (89.6)	0.197
Yes	1 (10.0)	18 (24.7)	30 (16.9)	5 (10.4)	
Re-excision					
No	9 (90.0)	68 (93.2)	166 (93.3)	47 (95.9)	0.871
Yes	1 (10.0)	5 (6.8)	12 (6.7)	2 (4.1)	
Radiotherapy					
No	0 (0)	10 (13.7)	21 (11.8)	18 (36.7)	<0.001
Yes	10 (100)	63 (86.3)	157 (88.2)	31 (63.3)	
Chemotherapy					
No	7 (70.0)	67 (91.8)	153 (86.0)	44 (89.8)	0.202
Yes	3 (30.0)	6 (8.2)	25 (14.0)	5 (10.2)	
Hormonal therapy					
No	5 (50.0)	28 (38.4)	78 (43.8)	19 (38.8)	0.774
Yes	5 (50.0)	45 (61.6)	100 (56.2)	30 (61.2)	
Benign histopathology					
No	10 (100)	70 (95.9)	166 (93.3)	41 (83.7)	0.052
Yes	0 (0)	3 (4.1)	12 (6.7)	8 (16.3)	

The median *Q*-score for 345 women who had answered the BREAST-Q™ postoperative questionnaire domain “Satisfaction with breasts” was 66 (IQR 57–80; range 12–100). Regarding “psychosocial well being”, the median was 82 (IQR 61–100; range 0–100). Only 207 women had answered enough of the questions in the domain “sexual well being” to be analysed. Median *Q*-score of this domain was 60 (IQR 48–79). For “adverse effects of radiation” (*n* = 338), median was 100 (IQR 89–100) and for “physical well being” (*n* = 339) it was 81 (69–92).

We chose to analyse BCCT.core results with the domains “satisfaction with breasts” and “psychosocial well being” since these were considered most likely to be affected by the aesthetic result and had an adequate questionnaire response rate.

In Table 3, quartiles of the *Q*-scores are related to the BCCT.core results. It can be observed that patients with a *Q*-score in the highest quartile more often had a good or excellent BCCT.core result, compared to those with a *Q*-score in the lowest quartile. In Table 4, BCCT.core results are related to *Q*-scores dichotomised with cut-off at the median. The patients with a fair/poor result on BCCT.core had lower *Q*-scores both concerning satisfaction with breasts (cut-off 66) with an OR of 3.4 (CI 1.7–6.8) and concerning psychosocial well-being (cut-off 82) with an OR of 2.2 (CI 1.1–4.2). The results remained statistically significant in the age-adjusted model. When also adjusting for the factors having a statistically significant association with result on BCCT.core in Table 2, i.e., BMI, breast size preoperatively, and radiotherapy, the OR for satisfaction with breasts was 2.6 (CI 1.2–5.4) and for psychosocial well-being it was 2.0 (CI 1.0–4.2).

**Table 3:** *Q*-score (quartiles) related to BCCT.core results (dichotomized and in four groups)

	Breast-Q™			
	Satisfaction with breasts			
	≤57 *n* (%)	58–66 *n* (%)	67–80 *n* (%)	>80 *n* (%)
BCCT.core result				
Good/excellent	33 (33.7)	34 (44.2)	52 (55.3)	44 (57.9)
Fair/poor	23 (23.5)	13 (16.9)	10 (10.6)	5 (6.6)
Missing	42 (42.9)	30 (39.0)	32 (34.0)	27 (35.5)
BCCT.core result				
Excellent	3 (5.4)	4 (8.5)	12 (19.4)	12 (24.5)
Good	30 (53.6)	30 (63.8)	40 (64.5)	32 (65.3)
Fair	19 (33.9)	13 (27.7)	9 (14.5)	4 (8.2)
Poor	4 (7.1)	0 (0)	1 (1.6)	1 (2.0)

	Breast-Q™			
	Psychosocial well-being			
	≤59 *n* (%)	60–82 *n* (%)	83–99 *n* (%)	100 *n* (%)
BCCT.core result				
Good/excellent	27 (33.3)	45 (48.4)	18 (46.2)	71 (55.5)
Fair/poor	16 (19.8)	16 (17.2)	6 (15.4)	12 (9.4)
Missing	38 (46.9)	32 (34.4)	15 (38.5)	45 (35.2)
BCCT.core result				
Excellent	3 (7.0)	8 (13.1)	3 (12.5)	17 (20.5)
Good	24 (55.8)	37 (60.7)	15 (62.5)	54 (65.1)
Fair	13 (30.2)	16 (26.2)	4 (16.7)	11 (13.3)
Poor	3 (7.0)	0 (0)	2 (8.3)	1 (1.2)

**Table 4:** *Q*-score (dichotomized) in relation to BCCT.core results

	Breast-Q™				
	Satisfaction with breasts				
	≤66 *n* (%)	>66 *n* (%)	OR (CI)	OR (CI)^a^	OR (CI)^b^
BCCT.core result					
Good/excellent	67 (65.0)	96 (86.5)	1	1	1
Fair/poor	36 (35.0)	15 (13.5)	3.4 (1.7–6.8)	3.4 (1.7–6.9)	2.6 (1.2–5.4)
BCCT.core result					
Excellent	7 (6.8)	24 (21.6)	1	1	1
Good	60 (58.3)	72 (64.9)	2.9 (1.2–7.1)	2.9 (1.2–7.2)	2.5 (1.0–6.5)
Fair	32 (31.1)	13 (11.7)	8.4 (2.9–24.4)	8.6 (2.9–25.0)	5.9 (1.9–18.5)
Poor	4 (3.9)	2 (1.8)	6.9 (1.0–45.6)	6.5 (1.0–43.8)	4.9 (0.7–36.1)

	Breast-Q™				
	Psychosocial well-being				
	≤82 *n* (%)	>82 *n* (%)			
BCCT.core result					
Good/excellent	72 (69.2)	89 (83.2)	1	1	1
Fair/poor	32 (30.8)	18 (16.8)	2.2 (1.1–4.2)	2.2 (1.1–4.2)	2.0 (1.0–4.2)
BCCT.core result					
Excellent	11 (10.6)	20 (18.7)	1	1	1
Good	61 (58.7)	69 (64.5)	1.6 (0.7–3.6)	1.6 (0.7–3.6)	1.6 (0.7–3.7)
Fair	29 (27.9)	15 (14.0)	3.5 (1.3–9.2)	3.4 (1.3–9.1)	3.3 (1.2–9.6)
Poor	3 (2.9)	3 (2.8)	1.8 (0.3–10.6)	1.7 (0.3–10.2)	1.5 (0.2–9.6)

^a^Adjusted for age

^b^Adjusted for age, BMI, breast volume, and radiotherapy

## Discussion

In this study, the results of the BCCT.core were excellent in 16% of cases, good in 57%, fair in 24%, and poor in 3%. A poor/fair result increased the risk of having *Q*-scores below median regarding satisfaction with breasts [(median 66) OR 3.4 (CI 1.7–6.8)] as well as with psychosocial well-being [(median 82) OR 2.2 (CI 1.1–4.2)]. The results remained statistically significant in both the adjusted models. 

There are few previous studies to compare our results with, given that the BREAST-Q™ BCT module is relatively new. In 2016, O’Connell et al. presented the first study using the BCT module. The study population was 200 women who had undergone unilateral BCT one to six years prior to the start of the study. A median *Q*-score of 68 (IQR 55–80) was reported regarding satisfaction with breasts and 82 (63–100) regarding psychosocial well-being [21]. These results are nearly identical to our results, indicating the possibility of *Q*-score reference values to be formed.

The BCCT.core results in our study can be compared to the long-term (3 years) follow-up results of 356 patients, who had undergone BCT, presented 2015 in a study by Hennigs et al. In their study cohort, the results of BCCT.core were excellent in 62 cases (17.4%), good in 181 (50.8%), fair in 101 (28.4%), and poor in 12 cases (3.4%), i.e., very similar to the results of our study [22]. In 2014 Haloua et al. reported BCCT.core results of excellent in 10 cases (9%), good in 54 (50%), fair in 34 (31%) and poor in 11 cases (10%) in a study cohort who had undergone BCT at least one year before the evaluation [23].

Kim et al. analysed outcome after latissimus dorsi flap reconstruction in 64 patients in a pilot study in 2015 [24]. With linear regression analysis, they could report an association between better BCCT.core results and higher *Q*-scores regarding satisfaction with breasts (*R* ^2^ = 0.070; *p* = 0.039) as well as with psychosocial well-being (*R* ^2^ = 0.085; *p* = 0.023). In our study, we showed an association between poor/fair BCCT.core results a year after BCT and lower *Q*-scores several years later (median 5.5 years). Whether the aesthetic result has changed during this time period is not known. Some studies have shown a long-term deterioration of the aesthetic result [25, 26] especially after aggressive radiotherapy. However, these studies are based on study populations treated decades ago and might not be applicable to patients treated today with more modern radiotherapy techniques. In 2017, Soror et al. compared aesthetic outcome evaluated by a panel of observers in 114 patients treated with accelerated partial breast irradiation after 3–4 weeks postoperatively to the result after approximately 3.5 years. In their material, about 60% had the same score, 36% had better, and 4% had worse scores [27]. In a study by Heil et al., aesthetic status, evaluated by patients completing the breast cancer treatment outcome scale (BCTOS) questionnaire, did not significantly differ from shortly after surgery compared to after one year [28]. Similarly using the BCTOS, Hennigs et al. reported that patients unsatisfied with aesthetic status shortly after surgery were likely to remain unsatisfied after 2–6 years [29].

### Limitations

Since the BREAST-Q™ BCT module had not been developed when our study was initiated, no preoperative assessment of quality of life by this method could be retrieved for comparison, even though it would have been valuable to have a baseline measurement.

There is the potential bias between responders and non-responders when administering follow-up questionnaires by mail. We have tried raising the response rate by two reminders by mail, and have reached a percentage of 76%, which we consider to be an acceptable response rate. In Table 1, baseline characteristics of the study group with BCCT.core results and completed BREAST-Q™ available can be compared to those of the entire study population and they show very similar results. We consider that the risk of any major selection bias is low.

The photographs in this study were not taken in a standardised way, i.e., with specific light setting and at a certain distance. However, in the literature published by the researchers behind BCCT.core, no such requirements are presented. The calibration of distance required was solved since NJ-distances were available. The measurement of NJ-distances has been validated previously [30].

## Conclusion

The results of aesthetic outcome using BCCT.core and that of HRQoL measured by the BREAST-Q™ BCT module in our study resonate well with results of other recent studies. This might contribute to the development of a credible reference range for clinicians using the postoperative module to compare with.

The association shown in this study, between a poor result one year after BCT evaluated with BCCT.core and lower *Q*-scores several years later measured with BREAST-Q™ BCT module strengthens the objective to use these evaluation instruments after BCT.

## Abbreviations

BCCT.core: Breast cancer conservative treatment. cosmetic results
BCS: Breast-conserving surgery
BCT: Breast-conserving therapy (BCS plus adjuvant radiotherapy)
BCTOS: Breast cancer treatment outcome scale
HRQoL: Health-related quality of life
IQR: Interquartile range
PROM: Patient-reported outcome measure

## Footnotes

1 Copyright notice: “Memorial Sloan-Kettering Cancer Center and the University of British Columbia™ 2006, All rights reserved”. Contact information and permission to use: MAPI Research Trust, Lyon, France. E-mail: PROinformation@mapi-trust.org; Internet: www.proqolid.org.
